# Cellular Localization of Kynurenine 3-Monooxygenase in the Brain: Challenging the Dogma

**DOI:** 10.3390/antiox11020315

**Published:** 2022-02-04

**Authors:** Korrapati V. Sathyasaikumar, Verónica Pérez de la Cruz, Benjamín Pineda, Gustavo Ignacio Vázquez Cervantes, Daniela Ramírez Ortega, David W. Donley, Paul L. Severson, Brian L. West, Flaviano Giorgini, Jonathan H. Fox, Robert Schwarcz

**Affiliations:** 1 Maryland Psychiatric Research Center, Department of Psychiatry, University of Maryland School of Medicine, Baltimore, MD 21228, USA; saikumar@som.umaryland.edu; 2 Neurobiochemistry and Behavior Laboratory, National Institute of Neurology and Neurosurgery “Manuel Velasco Suárez”, Mexico City 14269, Mexico; veped@yahoo.com.mx (V.P.d.l.C.); guigvace@gmail.com (G.I.V.C.); drmz_ortega@hotmail.com (D.R.O.); 3 Neuroimmunology Department, National Institute of Neurology and Neurosurgery “Manuel Velasco Suárez”, Mexico City 14269, Mexico; benjamin.pineda@innn.edu.mx; 4 Department of Veterinary Sciences, University of Wyoming, Laramie, WY 82071, USA; ddonley@harding.edu (D.W.D.); jfox7@uwyo.edu (J.H.F.); 5 Plexxikon Inc., South San Francisco, CA 94080, USA; seversonpaul@gmail.com (P.L.S.); bwest.cima@gmail.com (B.L.W.); 6 Department of Genetics and Genome Biology, University of Leicester, Leicester LE1 7JA, UK; fg36@le.ac.uk

**Keywords:** astrocyte, Huntington’s disease, kynurenine pathway, microglia, schizophrenia

## Abstract

Kynurenine 3-monooxygenase (KMO), a key player in the kynurenine pathway (KP) of tryptophan degradation, regulates the synthesis of the neuroactive metabolites 3-hydroxykynurenine (3-HK) and kynurenic acid (KYNA). KMO activity has been implicated in several major brain diseases including Huntington’s disease (HD) and schizophrenia. In the brain, KMO is widely believed to be predominantly localized in microglial cells, but verification in vivo has not been provided so far. Here, we examined KP metabolism in the brain after depleting microglial cells pharmacologically with the colony stimulating factor 1 receptor inhibitor PLX5622. Young adult mice were fed PLX5622 for 21 days and were euthanized either on the next day or after receiving normal chow for an additional 21 days. Expression of microglial marker genes was dramatically reduced on day 22 but had fully recovered by day 43. In both groups, PLX5622 treatment failed to affect *Kmo* expression, KMO activity or tissue levels of 3-HK and KYNA in the brain. In a parallel experiment, PLX5622 treatment also did not reduce KMO activity, 3-HK and KYNA in the brain of R6/2 mice (a model of HD with activated microglia). Finally, using freshly isolated mouse cells ex vivo, we found KMO only in microglia and neurons but not in astrocytes. Taken together, these data unexpectedly revealed that neurons contain a large proportion of functional KMO in the adult mouse brain under both physiological and pathological conditions.

## 1. Introduction

Kynurenine 3-monooxygenase (KMO) catalyzes the conversion of L-kynurenine to 3-hydroxykynurenine (3-HK) in the kynurenine pathway (KP), the major route of tryptophan degradation in eukaryotic organisms. While 3-HK is mainly known for generating reactive oxygen species and thereby causing oxidative damage, it is also able to scavenge free radicals and therefore has remarkable antioxidant properties. Importantly, because of its pivotal position in the KP, KMO is not only critical for 3-HK formation but controls the synthesis of several other biologically active KP metabolites, including kynurenic acid (KYNA), xanthurenic acid, 3-hydroxyanthranilic acid, xanthurenic acid, quinolinic acid, picolinic acid and cinnabarinic acid [1]. An NADPH-dependent enzyme located in the outer mitochondrial membrane [2,3,4] and linked to mitochondrial function [5], KMO is widely expressed in peripheral tissues, macrophages and monocytes [6,7]. Notably, as a number of KP metabolites are increasingly perceived to have considerable significance in normal brain function (see [8], for review), impaired KMO activity may play a substantive role in the pathophysiology of several neurological and psychiatric diseases [9,10,11,12]. For both conceptual and translationally relevant reasons, it is therefore essential to have a clear understanding of the cellular localization of KMO in the brain in health and disease.

**Figure 1:**
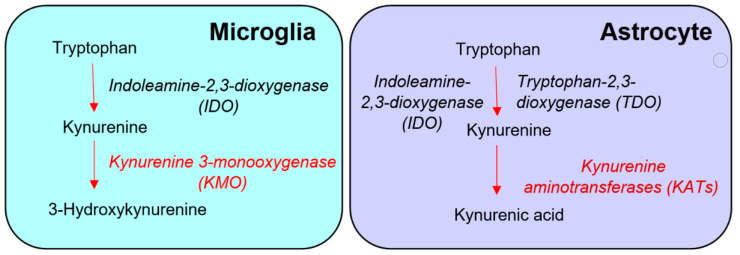
Conventional “glio-centric” view of the distinct de novo formation of 3-hydroxykynurenine and kynurenic acid in microglia and astrocytes, respectively.

Based almost entirely on studies of various cell types or immortalized cell lines in vitro, the prevailing view is that KMO in the central nervous system is predominantly localized in microglial cells (Figure 1) [13,14,15], and that inflammatory conditions greatly stimulate enzyme activity in these cells [13,16,17,18,19]. However, the presence of KMO has also been described in neurons and astrocytes in the rat brain [20]. KMO readily converts kynurenine to 3-HK in cultured fetal human neurons [21,22], and both KMO expression and function have been reported to be up-regulated in neurons in a mouse model of neuropathic pain [23]. Unfortunately, since state-of-the-art experimental in vivo tools have not been applied in this context so far, the present dogma that the major proportion of KMO in the mammalian brain is localized in microglial cells should therefore be considered with appropriate caution.

The present study was designed to examine the cellular localization of KMO in the mouse brain using two strategies. In a first approach, animals were treated for 3–4 weeks with the CSF1R inhibitor PLX5622. This procedure selectively eliminates more than 95% of microglial cells under both physiological and pathological conditions, though microglial re-population occurs rapidly once the treatment is discontinued [24,25,26]. Experiments were performed both in normal animals and in R6/2 mice, an established model of Huntington’s disease (HD; [27]), which shows microglial activation [28] and elevated KMO activity [29] in several brain areas. Because fluctuations in KMO activity play a critical role in the formation and function of the prominent neuromodulator KYNA, which is mainly synthesized in astrocytes (Figure 1) [30,31,32,33,34,35], we determined both enzyme activity and the brain tissue levels of 3-HK and KYNA in these studies.

In a complementary approach, we examined both *Kmo* expression and KMO activity in microglial cells, neurons and astrocytes that were acutely purified from healthy mouse forebrain using magnetic beads [36] and a neuron isolation kit [37].

## 2. Materials and Methods

### 2.1. Experimental Tools

3-HK, L-kynurenine (“kynurenine”), KYNA, NADPH, glucose-6-phosphate and glucose 6-phosphate dehydrogenase (GAPDH) were obtained from Sigma Aldrich (St. Louis, MO, USA). The KMO inhibitor Ro 61-8048 was a generous gift from Dr. Wolfgang Fröstl (Novartis, Basel, Switzerland). PLX5622 was provided by Plexxikon Inc. (South San Francisco, CA, USA) and formulated in standard AIN-76A chow (Research Diets, New Brunswick, NJ, USA).

MojoSort™ Buffer, anti-PE Nanobeads and anti-APC nanobeads, as well as PE-conjugated anti-mouse/human CD11b, APC-conjugated anti-mouse CD24 and Alexa Fluor 488 anti-mouse/rat MAP2 antibodies, were purchased from BioLegend (San Diego, CA, USA). Permeabilizing Solution 2 was obtained from BD Biosciences (San Diego, CA, USA). Rabbit anti-mouse kmO antibodies were provided by Avivas (San Diego, CA, USA). Mouse anti-mouse/rat GFAP-Cy3, mouse anti-mouse/rat/human NeuN and donkey anti-mouse IgG-Alexa 488 antibodies were purchased from AbCam (Boston, MA, USA), and mouse anti-mouse/rat/human Iba1 antibodies were from SantaCruz Biotechnology (Dallas, TX, USA). VECTASHIELD^R^ with 4′,6-diamidino-2-phenylindole (DAPI) was obtained from Vector Laboratories (Burlingame, CA, USA). Neuron isolation kits, PE-conjugated anti-mouse/rat GLAST antibodies and red blood cell lysis solution were provided by Miltenyi Biotech (San Diego, CA, USA), and anti-rabbit-Alexa Fluor 488 as well as anti-rabbit-Alexa Fluor 549 antibodies were purchased from Invitrogen (Carlsbad, CA, USA). All other chemicals were of the highest commercially available purity.

### 2.2. Animals

University of Maryland: Two-month-old male C57BL/6J mice, obtained from Jackson Laboratory (Bar Harbor, ME), were housed in a temperature-controlled, AAALAC-approved animal facility on a 12/12 h-light/dark cycle with unlimited access to food and water. The experimental protocol was approved by the Institutional Animal Care and Use Committee (IACUC) of the University of Maryland School of Medicine.

University of Wyoming: R6/2 HD mice (originally from Jackson Laboratory) were maintained by crossing R6/2 males with B6/CBA F1 females. Mice were given ad libitum access to food and water on a 12/12 h-light/dark cycle. Progeny were genotyped at 3 weeks of age using DNA obtained from tail tips, weaned at 3.5 weeks, and then assigned to cages based on HD gene status. The experimental protocol was approved by the IACUC of the University of Wyoming.

S.S.A. Ciudad de México: For the isolation of brain cells, 2-month-old mice (C57BL/6J) were obtained from the vivarium of the National Institute of Neurology and Neurosurgery (México City, México). The animals were housed in a temperature-controlled facility on a 12/12h-light/dark cycle with unlimited access to food and water. All procedures were carried out according to the National Institutes of Health Guide for the Care and Use of Laboratory Animals, and the local guidelines on the ethical use of animals from the Health Ministry of México.

### 2.3. PLX5622 Treatment In Vivo

University of Maryland: Animals were fed PLX5622 (1200 ppm in chow) or standard AIN-76A rodent chow ad libitum for 21 days. Either on the next day or after receiving normal chow for an additional 21 days, mice were euthanized in a CO_2_ chamber. The brains were then quickly removed, frozen on dry ice and stored at −80 °C until analysis.

University of Wyoming: Animals were fed PLX5622 (1200 ppm in chow) or standard AIN-76A rodent chow ad libitum for 28 days starting at 7.5 weeks of age. On the last day of treatment, mice were deeply anesthetized using a B-euthanasia solution and perfused with cold 0.9% (*w*/*v*) saline for 2 min via the left ventricle. The brains were then quickly removed, frozen on dry ice and stored at −80 °C until analysis.

### 2.4. Analyses of Brain Tissue Following In Vivo Treatment

#### 2.4.1. Expression of Microglial Markers and Kmo

Total mRNA was extracted from frozen brains using an RNeasy Mini Kit (Qiagen, Germantown, MD, USA), and cDNA was synthesized using iScript cDNA Synthesis kit (Bio-Rad, Hercules, CA, USA). RT-PCR for *AIF1, CSF1R, CX3CR1, Siglech, Tmem119, Kmo* and *GAPDH)* was performed using ABI TaqMan Expression Assays (Thermo-Fisher, San Francisco, CA, USA) (assay IDs: Mm00479862_g1, Mm01266652_m1, Mm00438354_m1, Mm00618627_m1, Mm01321343_m1 and Mm99999915_g1, respectively). Ct values were normalized to *GAPDH* and expressed as a percent of control.

#### 2.4.2. KMO Activity

Brain samples were weighed while frozen, homogenized (1:5, *w*/*v*) by sonication in ultrapure water, and further diluted 1:5 (*v*/*v*) in Tris-HCl buffer, pH 8.1, containing 10 mM KCl and 1 mM EDTA. The reaction mixture contained 80 µL of this solution, 1 mM NADPH, 3 mM glucose 6-phosphate, GAPDH (1 U) and 100 µM kynurenine in a total volume of 0.2 mL. Samples were incubated at 37 °C for 40 min in a shaking water bath. Blanks were obtained by adding the KMO inhibitor Ro 61-8048 (final concentration: 100 μM) to the incubation solution. The reaction was terminated by the addition of 50 µL of 6% perchloric acid. The samples were centrifuged (16,000× *g*, 15 min), and the resulting supernatant was diluted as needed. Twenty μL of the solution were then subjected to HPLC analysis of 3-HK, as described below.

#### 2.4.3. 3-Hydroxykynurenine (3-HK)

Twenty-five μL of 6% perchloric acid were added to 100 μL of the original brain homogenate. After thorough mixing, the precipitated proteins were removed by centrifugation (16,000× *g*, 15 min). Twenty μL of the resulting supernatant were applied to a 3 μm HR80 column (80 × 4.6 mm, Thermo-Fisher Scientific, Waltham, MA, USA), using a mobile phase consisting of 1.5% acetonitrile, 0.9% triethylamine, 0.59% phosphoric acid, 0.27 mM sodium EDTA and 8.9 mM heptane sulphonic acid and a flow rate of 0.5 mL/min. In the eluate, 3-HK was detected electrochemically (Eicom HTEC-500; San Diego, CA, USA) at an oxidation potential of +0.5 V. The retention time of 3-HK was ~11 min.

#### 2.4.4. Kynurenic Acid (KYNA)

Twenty μL of the solution used for the analysis of 3-HK (see above) were applied to a 3-µm ReproSil C18 column (100 mm × 4 mm; Dr. Maisch GmbH, Ammerbuch, Germany) to quantify KYNA. KYNA was isocratically eluted at a flow rate of 0.5 mL/min, using a mobile phase containing 50 mM sodium acetate and 3% acetonitrile (pH adjusted to 6.2 with glacial acetic acid). After post-column derivatization with 500 mM zinc acetate, delivered at a flow rate of 0.1 mL/min, KYNA was determined in the eluate by fluorometric detection (excitation: 344 nm, emission: 398 nm; Perkin-Elmer series 200; Waltham, MA, USA). The retention time of KYNA was ~18 min.

### 2.5. Isolation of Brain Cells

Mice were decapitated, and their brains were rapidly collected and washed with ice-cold phosphate-buffered saline (PBS). Forebrains were then immediately chopped, and 2 mL of accutase were added to achieve cell disaggregation. Next, 1 mL of fresh Dulbecco’s Modified Eagle Medium (DMEM), supplemented with 10% of fetal bovine serum (FBS), was added, and the cell suspension was filtered through pre-separation filters (70 µm; Miltenyi Biotec). After adjusting the volume to 10 mL with DMEM containing 10% of FBS, the samples were centrifuged (300× *g*, 10 min), and the supernatant was discarded. Subsequently, myelin sheaths and cell debris were removed using a debris removal solution (Miltenyi Biotec), and samples were centrifuged at 3000× *g* (10 min). The supernatant was discarded, and the pellets were washed with 15 mL of PBS and re-centrifuged (1000× *g*, 10 min). Following removal of the supernatant, addition of a red blood cell lysis solution (900 µL), and incubation for 10 min at 4 °C, 10 mL of PBS were added, and samples were centrifuged (300× *g*; 10 min). The resulting pellet was re-suspended in 200 µL of PBS and separated into two different pools to add PE-conjugated GLAST, PE-conjugated CD11b or APC-conjugated D24 antibodies (all in a final concentration of 1 µg/mL). After incubation in darkness at room temperature (30 min), cells were washed with 4 mL MojoSort buffer and centrifuged at 300× *g* (5 min). Cells were then re-suspended in 100 µL of MojoSort buffer with 10 µL of anti-PE nanobeads or anti-APC nanobeads, incubated for 15 min on ice, washed with 4 mL of MojoSort buffer, and again centrifuged at 300× *g* (5 min). For separation, 2.5 mL of MojoSort buffer were added, and each tube was placed for 5 min in a MojoSort magnet. Unattached cells were discarded. The remaining cells were suspended in 2.5 mL of MojoSort buffer and again placed in the magnet. This step was repeated twice for each tube. After cell separation, GLAST+, CD11b+ and CD24+ cells were collected to examine purity and yield, *Kmo* mRNA expression, and KMO activity.

Using a neuron isolation kit, the cell suspension obtained after disaggregation and debris removal (see above) was mixed with 20 µL of a non-neural cell biotin-antibody cocktail for 10 min in darkness at 4 °C. Cells were then washed with 1 mL of D-PBS/BSA buffer, centrifuged (300× *g*, 5 min) and re-suspended in 80 µL of D-PBS/BSA. Twenty µL of a solution containing anti-biotin microbeads were then added, and the mixture was incubated in darkness at 4 °C (10 min). Magnetic cell separation was performed with LS columns in a MidiMACS cell separator (Proteintech, San Diego, CA, USA). After isolation, neurons were treated with permeabilizing “Solution 2” as per the manufacturer’s instructions and incubated with Alexa Fluor 488 MAP2 primary antibody (1:100) for cell purity determination and measurement of KMO activity.

Following cell separation, the purity of astrocytes, microglia and neurons was determined using fluorescence-activated cell sorting (FACS) and Calibur flow cytometer software (Becton-Dickinson, San Jose, CA, USA), acquiring 10,000 total events. CellQuestPro and Flowjo software were used for analysis.

### 2.6. Analyses of Isolated Brain Cells

#### 2.6.1. Kmo Expression

Separated astrocytes, microglial cells and neurons were treated with Trizol (ThermoFisher) for RNA isolation. cDNA was obtained using the 1st Strand cDNA Synthesis Kit. RT-PCR for KMO and GAPDH was performed using KMO Mouse qPCR primer pair NM_133809, MP207238, GAPDH mouse qPCR primer pair MP 008084, and SensiFAST SYBR Master Mix Lo-Rox Kit. All tools used were obtained from OriGene (Rockville, MD, USA). CT values were normalized to *GAPDH*.

#### 2.6.2. FACS Analysis

Separated astrocytes, microglia and neurons, surface-stained with anti-GLAST-PE, CD11b-PE and CD24-APC antibodies, respectively (see above), were processed for intracellular staining. Using a permeabilizing solution, cells were incubated for 30 min with an anti kmO antibody (diluted 1:100) and were then washed once with PBS and incubated for 30 min with Alexa Fluor 488 (diluted 1:200) as a secondary antibody. FACS analysis was performed using a FACS Calibur flow cytometer (Becton-Dickinson), acquiring 10,000 total events. CellQuestPro and Flowjo software were used for analysis. Fluorescence minus One (FMO) control as well as unstained samples were used to set compensations and gating strategy, and data were analyzed using FlowJo v.10 software.

#### 2.6.3. Immunofluorescence

Purified astrocytes, microglial cells and neurons were fixed with 100% methanol and mounted on slides. Antibodies against CD11b, GFAP-Cy3 and NeuN (all diluted 1:200), were then used to verify respective cellular identities. As a secondary antibody for NeuN, Alexa 488 was used. Additionally, cells were incubated with anti kmO antibodies (Alexa 488 for microglia and astrocytes, and Alexa 549 for neurons). After incubation (30 min, room temperature) and 3 washes with PBS, each covering the slides for 5 min, slides were mounted using Vectashield with DAPI to counterstain the nucleus and were then sealed with nail polish. Images were taken using a Motic AE31Elite fluorescence microscope equipped with a CMOS camera (Photonfocus, Rockville, MD, USA).

#### 2.6.4. Quantitation of Cells

To determine the percentage of each cell type in the mouse forebrain, debris and red blood cells were removed from the original brain tissue homogenate, and cells were separated (see above). Respective antibodies (GFAP and GLAST for astrocytes, CD11b and Iba1 for microglia, and MAP2 for neurons) were then added to the suspension, and cells were quantitated by FACS as described above.

#### 2.6.5. KMO Activity

To determine KMO activity in separated astrocytes, microglia and neurons, 150 µL of isolated cell preparations (at least 1 × 10^5^ cells for each type) were diluted (1:2, v/v) in Tris-HCl buffer, pH 8.1, containing 10 mM KCl and 1 mM EDTA. Using the same reaction mixture described above, 80 µL of the samples were incubated at 37 °C for 2 h in a shaking water bath. Blanks were obtained using boiled samples (10 min). The reaction was terminated by the addition of 25 µL of 6% perchloric acid. After centrifugation (16,000× *g*, 10 min), the resulting supernatants were diluted as needed, and 100 μL were applied to a 3 μm HPLC column (Adsorbosphere C18, 100 mm × 4.6 mm, Fisher Scientific, Hampton, NH), using a mobile phase consisting of 1.5% acetonitrile, 0.9% triethylamine, 0.59% phosphoric acid, 0.27 mM sodium EDTA and 8.9 mM heptane sulphonic acid and a flow rate of 0.6 mL/min. 3-HK was detected electrochemically using a LC-4C detector (BAS, West Lafayette, IN; oxidation potential: +0.5 V at a range 1.0 nA and a filter of 0.10 Hz). The retention time of 3-HK was ~11 min.

### 2.7. Protein Determination

In all experiments, protein content was determined by adaptations of the method of Lowry et al. (1951), using bovine serum albumin as a standard [38].

### 2.8. Statistical Analyses

All data are expressed as the mean ± SEM. Statistical analysis was performed using the Kruskal–Wallis test with Dunn’s test for multiple pairwise comparisons. When comparing only two groups, a Mann–Whitney test was performed. Values of *p* < 0.05 were considered significant.

## 3. Results

### 3.1. Effect of PLX5622 Treatment in Healthy Mice

**Figure 2:**
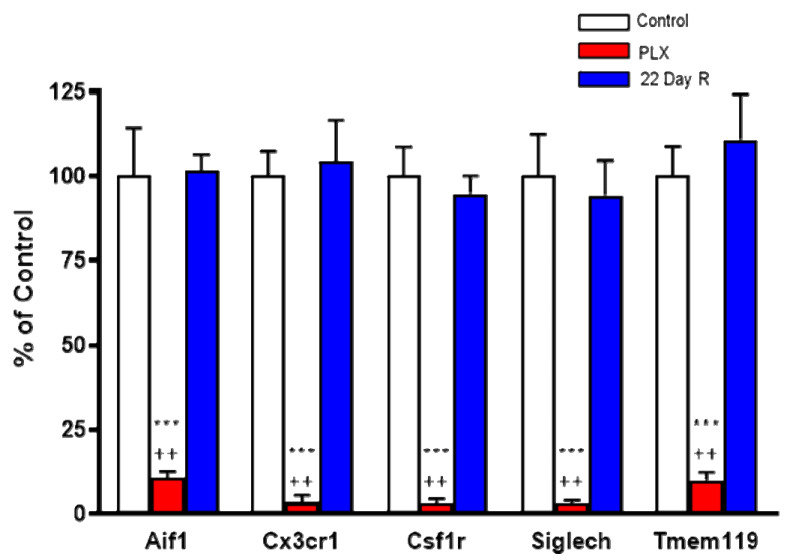
Effect of PLX5622 (PLX) treatment on microglial marker genes. Wild-type controls and PLX5622: *n* = 6 each; 22-day recovery (R): *n* = 3. Data (mean ± SEM) are expressed as a percentage of control values. See text for experimental details. *** *p* < 0.01 vs. control, ^++^ *p* < 0.05 vs. 22-day recovery (Kruskal–Wallis test with Dunn’s test for multiple pairwise comparisons).

Real time PCR analysis of the microglial marker genes *Aif1* (−89%), *Csf1r* (−97%), *Cx3cr1* (−97%), *Siglech* (−97%) and *Tmem119* (−90%) confirmed that daily administration of PLX5622 for 21 days caused the massive depletion of microglial cells in the forebrain of normal mice. However, the expression of these microglial markers was fully recovered 22 days after discontinuation of the treatment (Figure 2).

**Figure 3:**
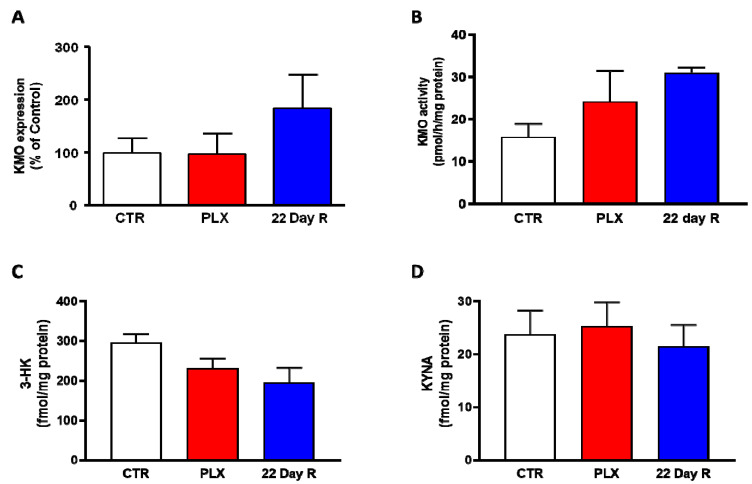
Effect of PLX5622 (PLX) on *Kmo* expression (**A**), KMO activity (**B**), 3-HK levels (**C**) and KYNA levels (**D**) in the forebrain of the same wild-type mice used to examine microglial marker genes (same *n*/group as in Figure 2). Data are the mean ± SEM. See text for experimental details. Kruskal–Wallis test, with Dunn’s test for multiple pairwise comparisons, revealed no significant group differences (*p* > 0.05).

In the same animals, neither *Kmo* expression nor KMO activity were significantly changed in the forebrain immediately after the treatment with PLX5622 or after a 22-day recovery period (Figure 3A,B). Forebrain 3-HK and KYNA levels, too, were not significantly affected by PLX5622 immediately after the treatment was terminated or 22 days later (Figure 3C,D).

### 3.2. Effect of PLX5622 Treatment in R6/2 Mice

**Figure 4:**
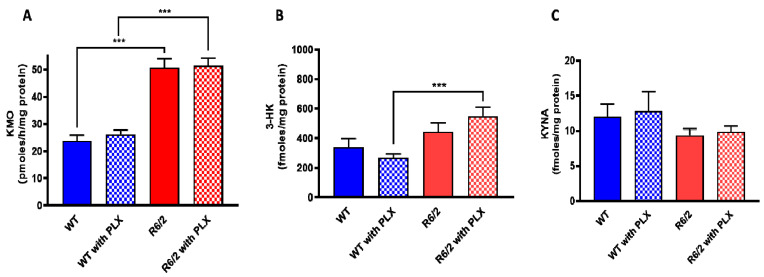
Effect of PLX5622 (PLX) on KMO activity (**A**), 3-HK (**B**) and KYNA (**C**) levels in the forebrain of wild-type (WT) and R6/2 mice. Data are the mean ± SEM (*n* = 10 per group). See text for experimental details. *** *p* < 0.001 (Kruskal–Wallis test with Dunn’s test for multiple pairwise comparisons).

We next examined the effect of prolonged PLX5622 treatment of R6/2 mice, a well-established animal model of HD which shows pronounced microglial activation [28]. As reported earlier [29] and confirmed here, KMO activity was significantly elevated in the brain in R6/2 mice compared to wild-type controls. However, PLX5622 had no effect on KMO activity, 3-HK and KYNA levels in the forebrain of either wild-type or R6/2 mice (all *p* > 0.05; Figure 4A–C). In agreement with the results obtained in normal mice (cf. Figure 3), PLX5622 also failed to influence KMO activity and the tissue concentrations of 3-HK and KYNA in the forebrain of the respective control animals (Figure 4A–C).

### 3.3. Kmo Expression and KMO Protein in Freshly Isolated Brain Cells

**Figure 5:**
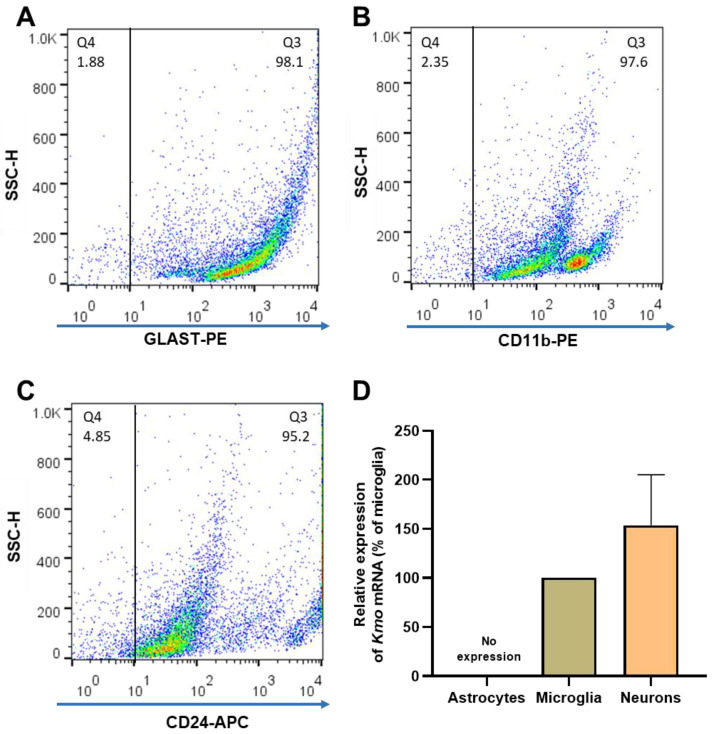
(**A**–**C**) Representative dot plots of side scatter height (SSC-H) vs. its corresponding marker, illustrating the purity of brain cells that were isolated from 6 separate mouse brains. After magnetic cell separation, the purity of astrocytes (GLAST-PE+), microglia (CD11b-PE+) and neurons (CD24-APC+) was evaluated by flow cytometry; (**D**) *Kmo* mRNA, determined by RT-qPCR. Data are the mean ± SEM. See text for experimental details.

In light of the unexpected failure to affect *Kmo* expression and activity, as well as 3-HK and KYNA levels, by experimentally depleting the vast majority of microglial cells in the brain in vivo, we decided to magnetically separate astrocytes, microglia, and neurons from whole mouse brain to examine the presence of KMO in each cell type. The purity of the freshly isolated cells was evaluated with specific surface markers for astrocytes (GLAST-PE), microglia (CD11b-PE) and neurons (CD24-APC). As illustrated in Figure 5A–C, the purity obtained using this procedure was 94.5 ± 1.0% for astrocytes, 92.5 ± 0.8% for microglia, and 95.7 ± 1.7% for neurons. Notably, microglia and neurons showed substantial *Kmo* expression, whereas no signal was detected in astrocytes (Figure 5D).

**Figure 6:**
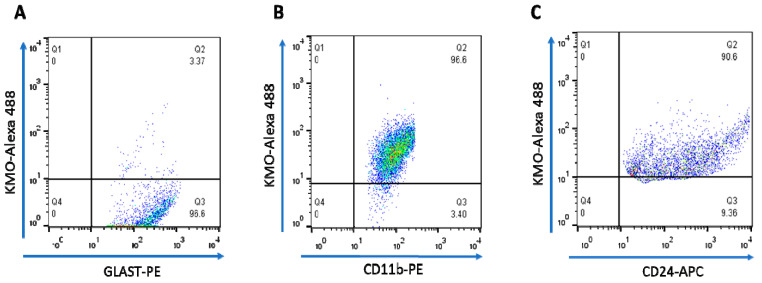
Dot plots illustrating intracellular staining of KMO protein in purified brain cells, assessed by FACS. The percentage of KMO+ cells in (A) astrocytes (GLAST-PE), (B) microglia (CD11b-PE) and (C) neurons (CD24-APC) is shown in Q2. See text for experimental details.

**Figure 7:**
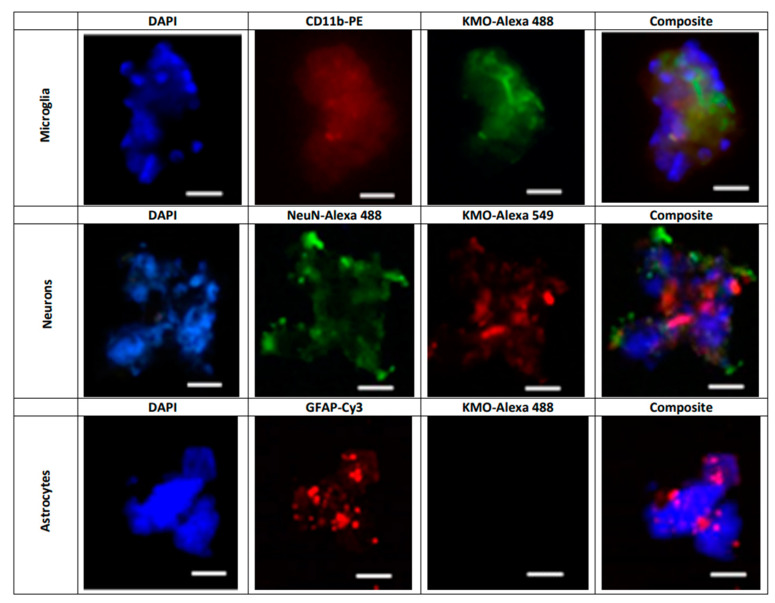
Representative images illustrating the presence of KMO protein in freshly isolated microglial cells (CD11b-PE: red), neurons (NeuN-Alexa 488: green) and astrocytes (GFAP-Cy3; red). Nuclei were stained with DAPI (blue). KMO was identified using anti-rabbit Alexa 488 in microglia and astrocytes, and anti-rabbit Alexa 549 in neurons, as secondary antibodies. See text for experimental details. Images were acquired at 40× magnification. Scale bars: 20 µm.

Next, KMO protein in these cells was examined by flow cytometry and immunofluorescence. In line with the *Kmo* mRNA expression data described above, double-staining revealed that less than 4% of GLAST+ cells, i.e., astrocytes, were KMO-positive (Figure 6A) whereas KMO was present in 95% of CD11b+ (Figure 6B) and 90% of CD24+ (Figure 6C) cells. Representative microscopic images of the cells, using anti-CD11b-PE, anti-NeuN-Alexa 488 and anti-GFAP-Cy3 antibodies to selectively stain microglia, neurons and astrocytes, respectively, are shown in Figure 7.

### 3.4. KMO Activity in Freshly Isolated Brain Cells

**Figure 8:**
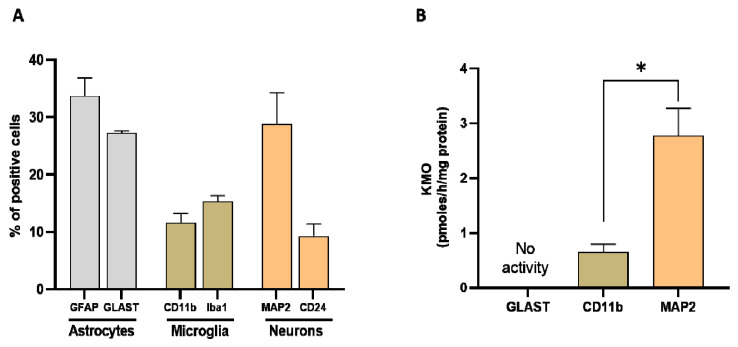
(**A**) Proportion of astrocytes, microglia, and neurons, assessed in whole mouse brain homogenate (*n* = 4–6). Cell suspensions were incubated with markers for astrocytes (GFAP and GLAST), microglia (CD11b and Iba1) and neurons (MAP2), respectively. See text for experimental details; (**B**) KMO activity in isolated cells (>90% purity). Data are the mean ± SEM. * *p* < 0.01 (Mann–Whitney test).

We next assessed the relative proportion of astrocytes, microglial cells and neurons in whole brain homogenate in order to determine their relative contribution to KMO activity in the normal mouse brain. Using specific markers for each cell type (GFAP and GLAST for astrocytes, CD11b and Iba1 for microglia, and MAP2 for neurons), the percentage of astrocytes was ~30% (GFAP: 34 ± 3%, GLAST: 27 ± 1%), the percentage of microglial cells was ~15% (CD11b: 12 ± 2%, Iba1: 15 ± 1%), and the percentage of neurons was 29 ± 5% (Figure 8A). KMO activity was detectable in microglia and neurons but not in astrocytes. Notably, specific enzyme activity was significantly higher in neurons than in microglial cells (Figure 8B).

## 4. Discussion

Using complementary experimental approaches, the present study was designed to examine the cellular localization of the KP enzyme KMO in the mouse brain. Using wild-type and HD model mice, we first determined both the expression and the activity of KMO and measured the levels of the KP metabolites 3-HK and KYNA, in brain tissue after selectively depleting microglia in vivo with the brain-penetrant CSF1R antagonist PLX5622 [24]. Unexpectedly, our results did not support the widely accepted view that a large majority of brain KMO is localized in microglial cells, and that these cells account for the increase in KMO activity seen under various pathological conditions. We then assessed the presence of KMO biochemically and microscopically ex vivo in microglial cells, astrocytes and neurons after they were acutely isolated from normal mice. While we confirmed previous studies in rats, gerbils and humans, which had shown that only very little—if any—KMO is contained in astrocytes [20,39,40], these experiments indicated that neurons may play a heretofore overlooked, substantive role in the regulation of KP function in brain physiology and pathology.

Due to the rapidly growing awareness of the multi-faceted neurobiological effects of several KP metabolites, and the insight that even relatively minor fluctuations in the brain levels of these compounds have biologically relevant functional consequences (see [1], for a recent comprehensive review), the pivotal location of KMO in the KP has made this enzyme an increasingly attractive focus of neuroscience research [9,10,12]. Notably, attention is not limited to the enzyme’s immediate product 3-HK, though the dual antioxidant and neurotoxic properties of this metabolite are intriguing and may be of considerable biological significance [41]. KMO also controls the fate of xanthurenic acid (XA), a transamination product of 3-HK [42] which is gaining attention in neurobiology because of its effect on glutamatergic neurotransmission [43,44] and its ability to modulate extracellular dopamine levels [45].

Notably, as demonstrated consistently in studies in animals, experimentally induced reductions in KMO activity raise the levels of the neuromodulator KYNA in the brain and elsewhere [30,31,34]. This functional connection between KMO and KYNA affects brain physiology [46,47,48,49] and may also have significant pathophysiological relevance since both impaired KMO activity and abnormal KYNA levels are seen in several neurological and psychiatric disorders including HD [50], epilepsy [51], schizophrenia [52,53,54], and bipolar disorder [35,55]. For this reason, we measured the brain tissue levels of both 3-HK and KYNA in our in vivo studies.

The realization that brain KYNA levels can be increased by KMO inhibition soon suggested attractive, novel therapeutic options [56,57,58]. Thus, reduced KMO activity would not only enhance the neuroprotective and anticonvulsant effects of KYNA [59], but at the same time decrease the synergistic adverse properties of 3-HK and its downstream product quinolinic acid, i.e., metabolites in the “neurotoxic branch” of the KP [60]. KMO inhibition was also envisioned to counter the detrimental effects of repeated stress or excessive immune activation, which have been proposed to play key roles in depressive disorders by increasing 3-HK and quinolinic acid levels in the brain [61,62,63,64,65,66]. It was soon understood, however, that elevated brain levels of KYNA—in line with the “Janus face” of the metabolite [67]—also have negative consequences, causing cognitive impairments due to interference with the function of both α7 nicotinic acetylcholine and NMDA receptors [68,69,70,71].

In mechanistic terms, the functional connection between KMO and KYNA is directly related to the fact that KMO (K_m_ for kynurenine: ~20 μM; [72]) is much more easily saturated by its substrate than kynurenine aminotransferases (KATs), i.e., KYNA’s biosynthetic enzymes (K_m_ values for kynurenine: >1 mM; [73]). In spite of the fact that KAT II, the main KAT responsible for the neosynthesis of rapidly mobilizable KYNA in the brain, is localized in astrocytes [32], i.e., in cells that do not contain KMO, rising concentrations of kynurenine therefore shift KP metabolism in vivo increasingly toward the production of KYNA [74]. This effect is commonly explained by the fact that kynurenine, which is the substrate of both KMO and KAT, is taken up separately by microglia [75] and astrocytes [76].

In view of this widely accepted “glio-centric” view of cerebral KP metabolism (cf. Figure 1), the failure of PLX5622 treatment to affect cerebral KMO activity, 3-HK and KYNA levels in healthy mice as well as in a mouse model of HD showing microglial activation, was unexpected and difficult to interpret. However, our data were in line with a recent report showing that PLX5622 treatment in mice does not prevent the sickness-inducing effects of acute inflammation, which are supposed to be causally related to microglial stimulation [77]. Without referring explicitly to KMO, the authors suggested plausibly that experimental depletion of microglial cells may have caused the stimulation of immune-activated genes in astrocytes or, less likely, enhanced trafficking of immune cells into the brain. Thus, although no KMO activity was detected in astrocytes in the present study, as yet undetermined compensatory astrocytic mechanisms may have accounted for the normal levels of 3-HK and KYNA seen in the brain of PLX5622-treated animals. Separately or in addition, compensatory changes in peripheral KP metabolism may be triggered by microglial ablation. Mechanisms may involve, for example, nitric oxide donors which inhibit the key upstream KP enzyme indoleamine-2,3-dioxygenase in macrophages but not in microglia [78,79], and may therefore affect entry of circulating kynurenines into the brain.

Since functionally significant activation of *neuronal* rather than microglial KMO accounts for depression-like behavior in a mouse model of neuropathic pain [23], a compensatory up-regulation of KMO in neurons appeared to be a possible alternative explanation of our in vivo results. In fact, neurons actively accumulate kynurenine [80], and the presence of KMO and KATs has been documented in a variety of mammalian neurons under both physiological and pathological conditions [20,21,22,81,82,83,84]. Our present observation that a *majority* of KMO in freshly isolated mouse brain cells was found in neurons was still unexpected, however. Although the cell isolation procedure, which is known to alter the gene expression profile of microglial cells and ought to be re-examined using a single-cell RNA-seq approach [85], may have affected our data in quantitative terms, these results imply that neurons play a far greater role in cerebral KP function than assumed.

While substantive species differences exist and must be taken into account [6,84], our new data indicate that the established, mostly glio-centric views of the nature of KP metabolism in the mammalian brain are too simplistic and should be re-visited. Of note in this context, selective neuronal ablation [74] as well as interference with astrocyte function [86] have distinct effects on cerebral KP metabolism in vivo but these observations, like the present study using PLX5622 to transiently incapacitate microglial cells, failed to provide a clear picture regarding the role(s) of KP-related cellular cross-talk in the brain. New, detailed approaches are therefore needed to elucidate the anatomical, biochemical and physiological features of KP metabolism, including the respective roles of receptors of KYNA and several other neuroactive KP metabolites, in distinct brain cells. Considering the complexities of cerebral KP function [1] as well as the critical role of peripherally derived kynurenine [87], special attention ought to be paid to additional cell types such as oligodendrocytes [88] and perivascular cells [89], and to the evaluation of brain region-specific heterogeneities. In view of the functionally relevant intricacies of the *Kmo* gene [35,53,90] and the recently described presence of functional KMO in astrocytomas [91], it seems especially relevant to assess the localization and function of the third kynurenine-degrading enzyme, kynureninase (K_m_: ~35 μM; [92]), in parallel with KMO and KATs in these experiments.

## 5. Conclusions

The present results suggest that a possible functional role of neurons in KMO-related events in the brain should be examined in detail. As cellular co-localization of KMO with other KP enzymes is likely to change both qualitatively and quantitatively under pathological conditions, deciphering the related functional implications, including effects on neurobiologically relevant redox processes, presents some of the most interesting and translationally relevant challenges. Importantly, future efforts should focus not only on events in the adult brain but consider that biologically significant differences in cerebral KMO function—and in brain KP metabolism in general—may exist in the pre- and early postnatal period and/or late in life (see [93] and [94] for reviews).

Eventually, these novel insights will have to be integrated into the larger role of the KP in physiology and pathology, with special attention not only to the individual, and remarkably complex, redox properties of kynurenine [95,96], 3-HK ([97,98] and KYNA [99,100], but also to the equally intriguing pro- and antioxidant properties of the downstream KP metabolites 3-hydroxyanthranilic acid, xanthurenic acid, quinolinic acid, picolinic acid and cinnabarinic acid (cf. Introduction). Inflammatory processes (including immunosuppression and immunotolerance [101,102,103,104], as well as investigations related to cellular energy status [105,106], neurodegenerative and neuroprotective processes [42,107,108], will deserve special emphasis in this respect. In view of the increasing realization that the KP has “a finger in every pie” [109], all these studies can be expected to have a significant and translationally relevant impact in the field.

## Data Availability

The datasets used in the current study are available from the corresponding author on reasonable request.
